# Analysis of the Effect of SNAI Family in Breast Cancer and Immune Cell

**DOI:** 10.3389/fcell.2022.906885

**Published:** 2022-07-08

**Authors:** Yifei Tu, Pengfei Fang, Long Zhang, Kewang Sun

**Affiliations:** ^1^ MOE Laboratory of Biosystems Homeostasis & Protection and Innovation Center for Cell Signaling Network, Life Sciences Institute, Zhejiang University, Hangzhou, China; ^2^ General Surgery, Cancer Center, Department of Breast Surgery, Zhejiang Provincial People’s Hospital (Affiliated People’s Hospital, Hangzhou Medical College), Hangzhou, China

**Keywords:** SNAI, breast cancer, immune cell, methylation, immunomodulator, drug

## Abstract

SNAI family members are transcriptional repressors that induce epithelial-mesenchymal transition during biological development. SNAIs both have tumor-promoting and tumor-inhibiting effect. There are key regulatory effects on tumor onset and development, and patient prognosis in infiltrations of immune cell and tumor microenvironmental changes. However, the relationships between SNAIs and immune cell infiltration remain unclear. We comprehensively analyzed the roles of SNAIs in cancer. We used Oncomine and TCGA data to analyze pan-cancer *SNAI* transcript levels. By analyzing UALCAN data, we found correlations between *SNAI* transcript levels and breast cancer patient characteristics. Kaplan–Meier plotter analysis revealed that SNAI1 and SNAI2 have a bad prognosis, whereas SNAI3 is the opposite. Analysis using the cBio Cancer Genomics Portal revealed alterations in SNAIs in breast cancer subtypes. Gene Ontology analysis and gene set enrichment analysis were used to analyze differentially expressed genes related to SNAI proteins in breast cancer. We used TIMER to analyze the effects of *SNAI* transcript levels, mutations, methylation levels, and gene copy number in the infiltration of immune cell. Further, we found the relationships between immune cell infiltration, SNAI expression levels, and patient outcomes. To explore how SNAI proteins affect immune cell, we further studied the correlations between immunomodulator expression, chemokine expression, and SNAI expression. The results showed that SNAI protein levels were correlated with the expression of several immunomodulators and chemokines. Through analysis of PharmacoDB data, we identified antitumor drugs related to SNAI family members and analyzed their IC50 effects on various breast cancer cell lines. In summary, our study revealed that SNAI family members regulate different immune cells infiltrations by gene copy number, mutation, methylation, and expression level. SNAI3 and SNIA1/2 have opposite regulatory effects. They all play a key role in tumor development and immune cell infiltration, and can provide a potential target for drug therapy.

## Introduction

Breast cancer is the most common tumor and has a high fatality rate worldwide (Siegel et al., 2021). The new cases of breast cancer worldwide surpassed that of lung cancer, and breast cancer became the cancer with the highest number of diagnoses in 2020. In the past year, there were approximately 19.3 million new cancer cases globally. Among these, there were approximately 2.3 million newly female breast cancer cases, accounting for 11.7% (Sung et al., 2021). The onset of breast cancer is related to numerous factors. Through whole-genome sequencing of breast cancer patients, numerous breast cancer-related genes, including genes of the *SNAI* family, have been identified.

The SNAIL family of zinc finger transcription factors consists of three members in vertebrates: SNAIL1 (encoded by *SNAI1*), SNAIL2 (encoded by *SNAI2*, also named SLUG), and SNAIL3 (encoded by *SNAI3*, also named SMUC) (de Herreros et al., 2010). All three SNAI proteins are expressed in the mammary glands and function as transcriptional repressors in physiological and pathological states (Peinado et al., 2007). SNAI1 is involved in the induction of epithelial-to-mesenchymal transition (EMT) and embryonic mesoderm formation and maintenance (Puisieux et al., 2014; Baulida et al., 2019). Mechanistically, it transcriptionally represses CDH1, which leads to loss of E-cadherin expression, EMT induction, and tumor cell invasion (Cano et al., 2000). EMT is important in malignant transformation and tumor progression (Larue and Bellacosa, 2005). SNAI2 is also involved in EMT induction (Bolos et al., 2003). It plays an essential role in TWIST-induced EMT by repressing BRCA2 expression and E-cadherin/CDH1 gene transcription (Tripathi et al., 2005; Caramel et al., 2013). SNAI1/2 are related to the malignant biological properties of tumor cells. Clinical data indicate that these two proteins are positively correlated with a high rate of metastasis and poor prognosis in various malignant tumors (Shin et al., 2012). Compared to SNAI1 and SNAI2, SNAI3 is less well studied. The function of SNAI3 in tumor progression is unknown, but it has been reported that it may be related to survival (Madden et al., 2014). SNAI3 has five zinc finger domains and is structurally similar to SNAI1 and SNAI2.

Several studies have demonstrated that SNAI family members are associated with patient prognosis in various tumor malignancies. We used different databases to comprehensively analyze the correlation between SNAIs and breast cancer to identify its complexity. In this study, we analyzed *SNAI* transcript levels, promoter methylation levels, and gene alterations in breast cancer, and their relationships with immune cell infiltration and patient prognosis. In addition, we analyzed the tumor killing effects of small-molecule drugs related to SNAI proteins. Our research shows that the SNAI family members play important roles in breast cancer and immune cell infiltration, and may provide some help for the early diagnosis of breast cancer and the development of new drugs.

## Methods

### Pan-Cancer Analysis

By using Oncomine database to compare the expression levels of SNAI members among various cancer types. The *p*-value was set at 1E-4, and the fold change was set at 2.

### Interactive Gene Expression Profiling Analysis

Using the Gene Expression Profiling Interactive Analysis (GEPIA) tool to profile and compare SNAI expression in various breast cancer stages, which was developed based on The Cancer Genome Atlas (TCGA) and Genotype-Tissue Expression (GTEx) databases (Tang et al., 2017).

### UALCAN Database Analysis

Using UALCAN portal (Chandrashekar et al., 2017) to analyze the clinical characteristics of breast cancer patients correlate with SNAI expression profiles and SNAI promoter methylation status.

### Kaplan–Meier Plotter Analysis

The relationships between SNAI expression levels and the prognosis of breast cancer patients, was analyzed using the Kaplan–Meier plotter (Gyorffy et al., 2010). In total, 2032 patients were divided in two groups according to the median SNAI expression level. The two groups were compared for overall survival (OS), distant metastasis-free survival (DMFS), relapse-free survival (RFS), and progression-free survival (PFS).

### cBio Cancer Genomics Portal (cBioPortal) Analysis

*SNAI* alterations in breast cancer subtypes were analyzed using the breast invasive carcinoma dataset (TCGA, PanCancer Atlas) (Cerami et al., 2012), which includes data from 1,084 samples.

### LinkedOmics Database Analysis

LinkedOmics is available portal containing data on 32 cancer types and 10 clinical proteomics tumor analysis consortium (CPTIC) cancer cohort (Vasaikar et al., 2018). It was used to analysis Kyoto Encyclopedia Genes and Genomes (KEGG) pathway and Gene Ontology (GO) according to biological process, cellular component and molecular function.

### TIMER Analysis

Using the TIMER web server (Li et al., 2017) analyze different immune cell infiltrations, including B cells, CD8^+^ T cells, CD4^+^ T cells, macrophages, neutrophils, and dendritic cells, in breast cancer. The correlations between gene expression, mutation, copy number alterations and immune cell infiltrations were analyzed.

### Gene Set Cancer Analysis

Using the Gene Set Cancer Analysis database (Liu et al., 2018) analyze the relationships between *SNAI* methylation levels and immune cell infiltrations in breast cancer.

### PharmacoDB Analysis

The PharmacoDB database was used to analyze the IC50 effects of different drugs on various cancer cells. The cutoffs used were an absolute value of correlation >0.1 and *p* < 0.05.

### TCGA Pan-Cancer Data Analysis

The Xena browser was used to analyze RNA-seq data, clinical information, and stemness scores based on mRNA and immune subtype data for various cancer types. These samples were used to compare gene expression profiles and any-omics data within a gene or transcript between tumors and normal tissues.

### TISIDB Analysis

TISIDB is used for interactions between tumor and immune system that integrates multiple heterogeneous data types (Ru et al., 2019). The database was used to analyze correlations between SNAI expression levels and immune cell infiltration as well as immunomodulator and chemokine levels in breast cancer.

## Results

### Pan-Cancer Analysis of SNAI Expression

**FIGURE 1:**
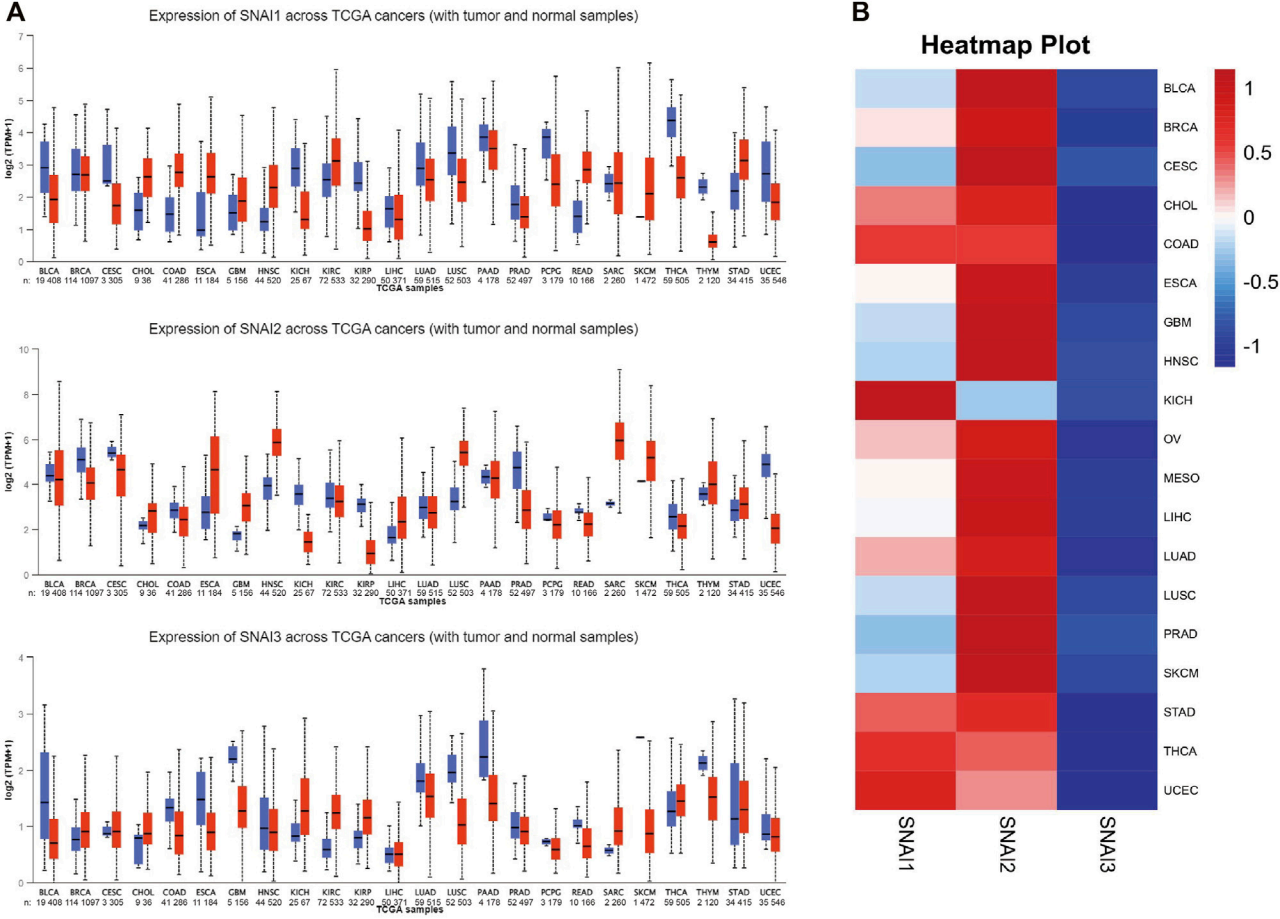
Transcript levels of SNAI zinc finger genes in various types of cancers **(A)** Transcript levels of *SNAI1*, *SNAI2*, and *SNAI3* in tumor tissues compared with normal tissues for various cancers **(B)** Comparison of transcript levels of *SNAI1*, *SNAI2*, and *SNAI3* among various cancer types based on The Cancer Genome Atlas data.

To study the roles of SNAIs expression levels in tumors, we retrieved clinical data and RNA-seq data of 33 tumors and non-cancer tissues. The data showed that SNAI expression differs among different types of cancer (Figure 1A). Transcript levels of SNAI1 and SNAI2 were increased in most types of tumors when compared with non-cancer tissues. SNAI3 expression was decreased in various tumor types. In breast cancer, SNAI1 and SNAI2 expression was high, whereas SNAI3 expression was low (Figure 1B).

### *SNAI* Transcript Levels in Breast Cancer

**FIGURE 2:**
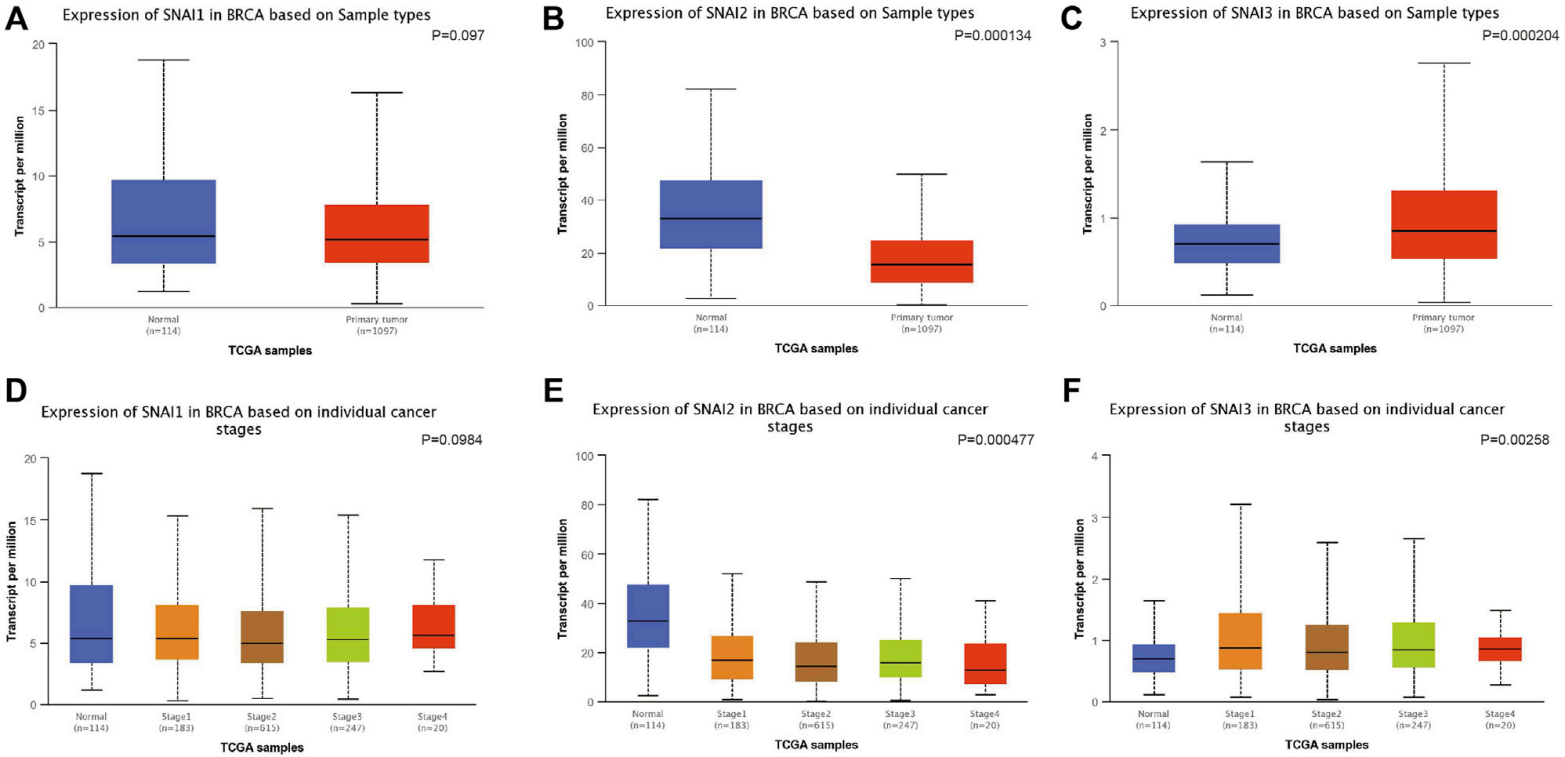
Correlations between SNAI expression and breast cancer. Transcript levels of *SNAI1* **(A)**, *SNAI2* **(B)**, and *SNAI3* **(C)** in breast cancer tissues compared with normal tissues. Transcript levels of *SNAI1* **(D)**, *SNAI2* **(E)**, and *SNAI3* **(F)** in various stages of breast cancer.

We used UALCAN to study the SNAIs expression in breast cancer cells. *SNAI1* expression level was no significantly different in primary cancer cells and non-cancer tissues (Figure 2A). However, *SNAI2* and *SNAI3* expression were downregulated and upregulated, respectively, in primary cancer cells (Figures 2B,C). These results were not consistent with previous findings. Due to the use of different algorithms, the resulting results would also be different direction. These results may indicate that SNAI proteins are more relevant in the breast cancer metastasis. Further, we found the SNAI proteins expression levels did not significantly differ according to the stage of breast cancer (Figures 2D–F).

The relationships between SNAI expression levels and breast cancer patient characteristics were evaluated using TCGA data. We discovered the expression level of SNAI1 was lower in men than in women and tended to be higher in Asians than in Americans and Africans (Supplementary Figures S1A, B). We found no obvious difference in SNAI1 levels between histological types (Supplementary Figure S1C). *SNAI1* transcript levels and *TP53* mutation status correlation found through study; patients with *TP53* mutation have higher *SNAI1* transcript levels than healthy people (Supplementary Figure S1D). At the same time, we also further analyzed the expression levels of SNAI2 and SNAI3 in breast cancer. These results indicated that SNAI transcript levels are related to the *TP53* mutation in patients with breast cancer.

### Survival Analysis

**FIGURE 3:**
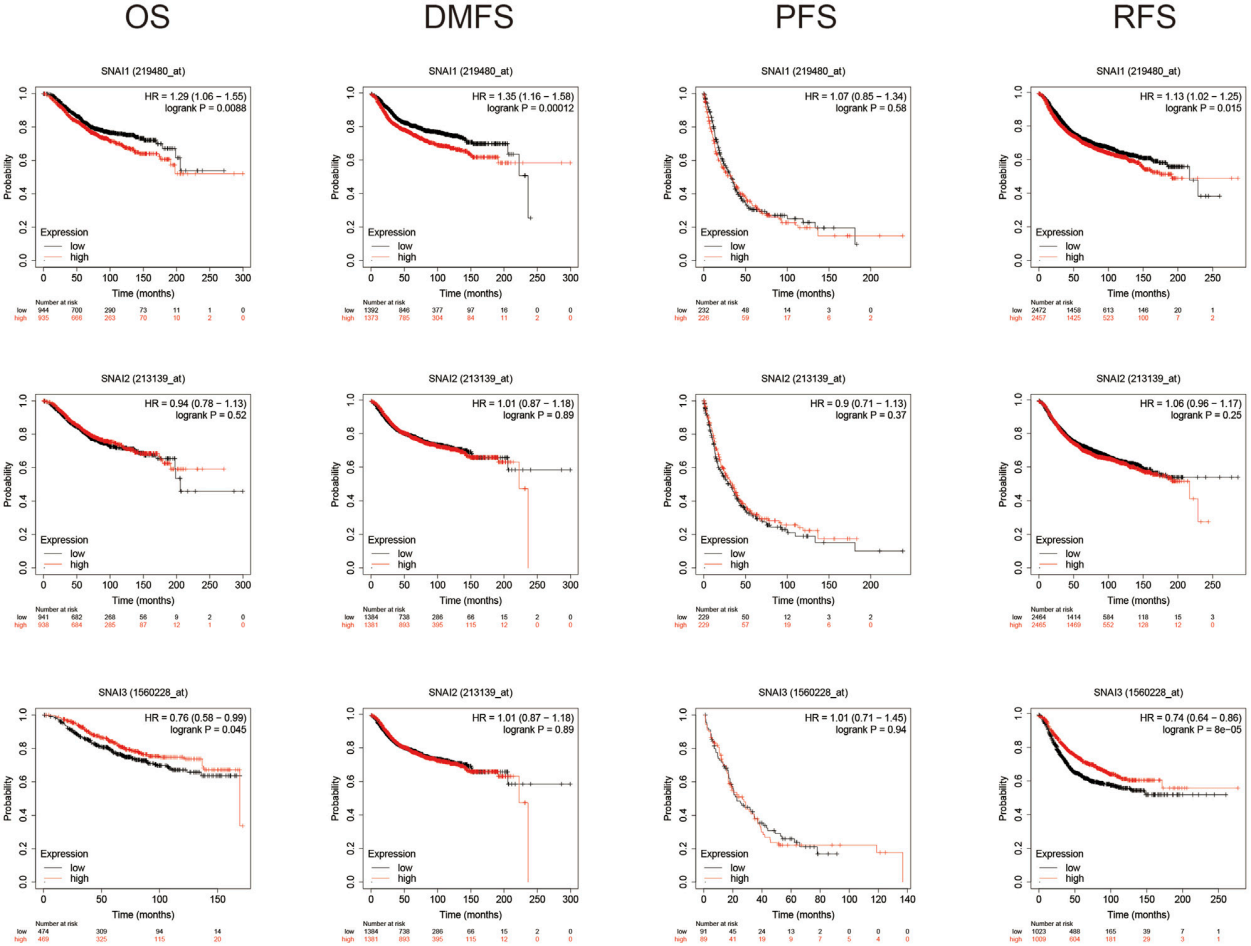
Prognostic value of *SNAI* transcript levels in breast cancer patients. Breast cancer patients were classified into two groups based on the median expression levels of *SNAI1*, *SNAI2*, or *SNAI3*. Overall survival (OS), distant metastasis-free survival (DMFS), progression-free survival (PFS), and relapse-free survival (RFS) were compared between patients with high SNAI expression and those with low SNAI expression.

Using Kaplan–Meyer plotter analysis analyzed the relationships between SNAI expression levels and patient prognosis. Results showed higher SNAI1 expression levels in patients had shorter OS, DMFS, and RFS. Higher SNAI2 expression had shorter DMFS and RFS, whereas higher SNAI3 expression had longer OS and RFS (Figure 3). These results indicated that SNAI1 and SNAI2 are associated with a poor prognosis and may play key effects in the DMFS and RFS, whereas SNAI3 seems to have an opposite effect and is related with a good prognosis.

### *SNAI* Alterations in Various Cancers

We used cBioPortal to analyze SNAI alterations, including fusions, amplifications, mutations, structural variants, deep deletions, and multiple alterations, in various cancer subtypes. SNAI1 and SNAI2 showed similar alteration tendencies in different subtypes, whereas SNAI3 showed different. The highest frequencies of SNAI alteration types among tumors were colorectal adenocarcinoma, invasive breast carcinoma, endometrial carcinoma, and epithelial ovarian carcinoma (Supplementary Figure S2A). For SNAI1, amplification ranked first among all alteration types in colorectal adenocarcinoma (6.73%), esophageal adenocarcinoma (4.4%), and invasive breast carcinoma (3.41%). For SNAI2, amplification was the most frequent alteration, with 4.43% on breast invasive carcinoma, and of 3.06% in endometrial carcinoma. In contrast, for SNAI3, deep deletion was the most frequent alteration, with a percentage of 2.4% in epithelial ovarian carcinoma and of 2.49% in invasive breast carcinoma. SNAI2 showed the highest number of alterations among the three SNAIs (Supplementary Figure S2B). When we analyzed the mutation sites in the SNAI genes, we found that the major mutation sites are located near the C2H2 domain (Supplementary Figure S3A). When we simulated the mutation sites of 3D protein structure, we found that the major mutations have quite a large impact on the protein structure (Supplementary Figure S3B).

### Analysis of DEGs Correlated With *SNAI* Genes in Breast Cancer

**FIGURE 4:**
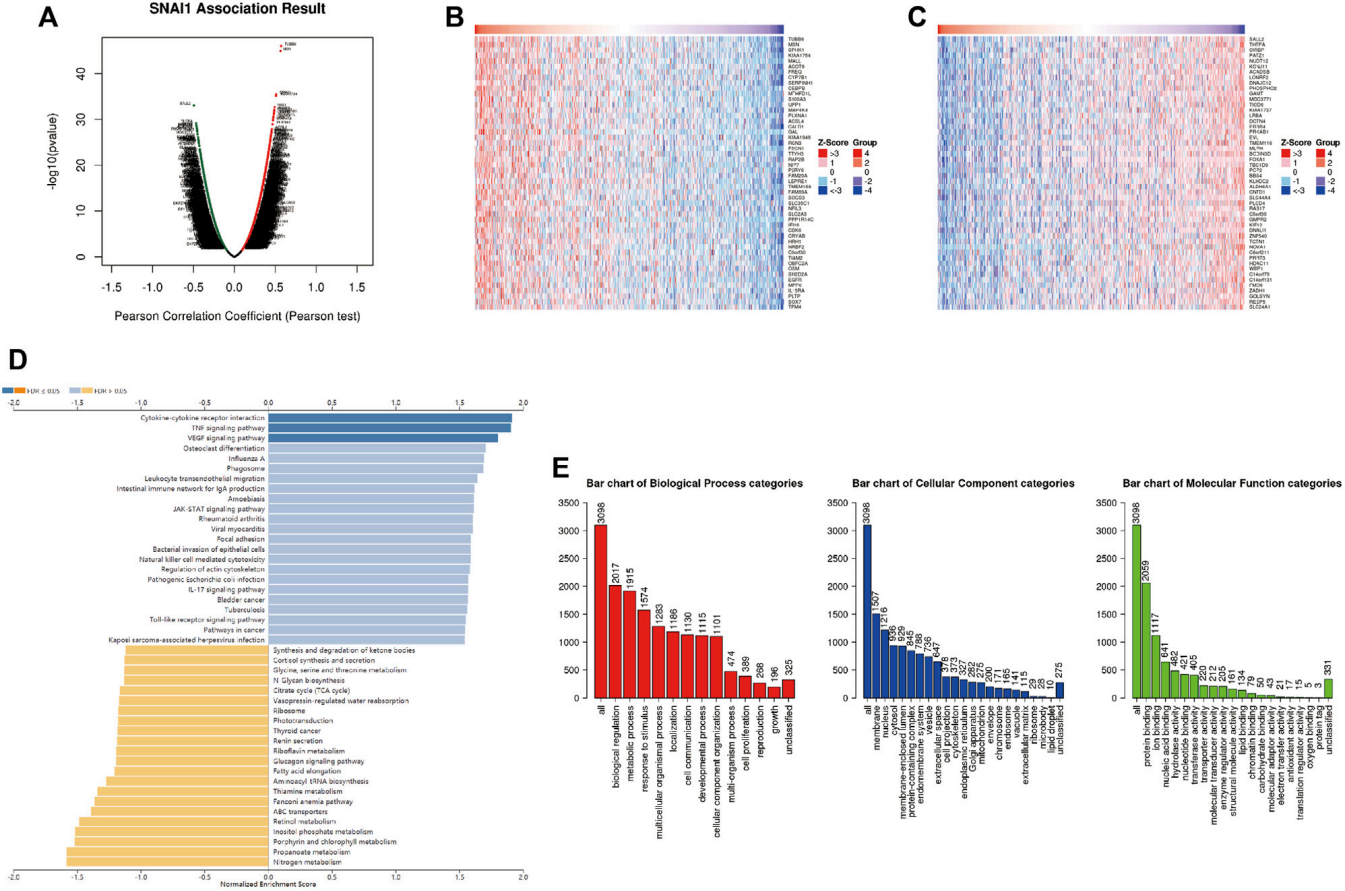
Analysis of differentially expressed genes (DEGs) in correlation with SNAI1 in breast cancer **(A)** Volcano plot showing the up- and downregulated genes correlated with SNAI1 expression (Pearson test). Significantly positively correlated **(B)** and negatively correlated **(C)** genes are shown in heatmaps **(D)** Kyoto Encyclopedia Genes and Genomes (KEGG) pathway analysis of the significant DEGs in correlation with SNAI1 **(E)** Gene ontology (GO) analysis of the significant DEGs in correlation with SNAI1.

LinkedOmics was used to analyze DEGs related with *SNAI1* in breast cancer (Figure 4A). We showed the 50 genes that most positively and negatively related to *SNAI1* in the form of heatmaps (Figures 4B,C). Significant DEGs were subjected to KEGG pathway enrichment analysis by GSEA (Figure 4D). We found that the TNF signaling pathway was significantly upregulated, whereas nitrogen metabolism, propanoate metabolism, and aminoacyl-tRNA biosynthesis were significantly downregulated in breast tumors. We next classified the DEGs using GO analysis (Figure 4E). The three most enriched biological process terms were biological regulation, metabolic process, and response to stimulus. The three most enriched cellular component terms were membrane, nucleus, and cytosol. The most enriched molecular function terms was protein binding. Classification and enrichment for DEGs correlated with SNAI2 and SNAI3 are shown in Supplementary Figures S4, S5. GSEA showed that genes correlated with autophagy, JAK-STAT signaling, TNF signaling, and VEGF signaling were significantly enriched in SNAI1-high patients. In SNAI2-high patients, genes correlated with autophagy, hippo signaling, EGFR tyrosine kinase inhibitor resistance, and TGF-β signaling were significantly enriched. In SNAI3-high patients, genes correlated with natural killer cell-mediated cytotoxicity, NF-κB signaling, T cell receptor signaling, and chemokine signaling were significantly enriched. These results show that the SNAI family is related to variety of signaling pathways, and may change the fate of cells by regulating different signal pathways.

### *SNAI* Promoter Methylation in Breast Cancer

Gene promoter methylation plays an important role in the regulation of gene expression. We conducted UALCAN analysis to study SNAI1 promoter methylation and their correlation with patient characteristics. In all clinical stages of breast cancer, we found the level of SNAI1 promoter methylation was lower in tumor tissues than in non-cancer tissues, and similar findings were made for subclass, TP53 mutation, and histological type (Supplementary Figure S6). MEXPRESS data was used to analyze the SNAI promoter methylation levels in breast cancer, and got similar results. These results indicated that methylation levels of the SNAI promoters may account for the different effects of SNAI proteins on outcomes.

### Correlation Between SNAI Expression Levels and Immune Infiltrate Abundance in Breast Cancer

**FIGURE 5:**
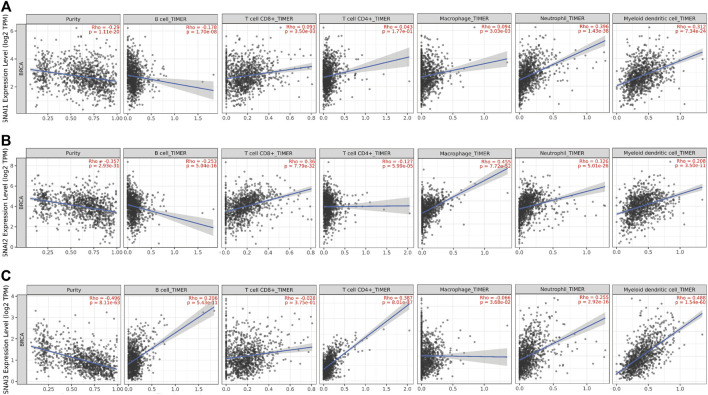
Correlations between differentially expressed SNAIs and immune cell infiltration in breast cancer. Correlations between transcript levels of **(A)** *SNAI1* **(B)** *SNAI2*, and **(C)** *SNAI3* and the infiltration of B cells, CD8^+^ T cells, CD4^+^ T cells, macrophages, neutrophils, and dendritic cells in breast cancer are shown.

SNAI1 proteins regulate the immune cells infiltration; however, the relationships between SNAI expression levels and immune cell infiltration in breast cancer are unclear. TIMER data was used to study the correlations between SNAI expression levels and the infiltration of six types of immune cells (i.e., B cells, CD8^+^ T cells, CD4^+^ T cells, macrophages, neutrophils, and dendritic cells) in breast cancer. We found the SNAI1 expression level was positively correlated with the infiltration of CD8^+^ T cells (R = 0.093, *p* = 3.50E-03), CD4^+^ T cells (R = 0.043, *p* = 1.77E-01), macrophages (R = 0.094, *p* = 3.03E-03), neutrophils (R = 0.396, *p* = 1.43E-38), and dendritic cells (R = 0.312, *p* = 7.34E-24), whereas it was negatively correlated with B cell infiltration (R = –0.178, *p* = 1.70E-08) (Figure 5A). SNAI2 was positively related with the infiltration of CD8^+^ T cells (R = 0.36, *p* = 7.79E-32), macrophages (R = 0.455, *p* = 7.72E-52), neutrophils (R = 0.326, *p* = 5.01E-26), and dendritic cells (R = 0.208, *p* = 3.50E-11), whereas it was negatively correlated with B cell infiltration (R = −0.253, *p* = 5.04E-16) and CD4^+^ T cells infiltration (R = –0.127, *p* = 5.99E-05) (Figure 5B). SNAI3 was positively correlated with the infiltration of B cells (R = 0.206, *p* = 5.43E-11), CD4^+^ T cells (R = 0.387, *p* = 8.01E-37), neutrophils (R = 0.255, *p* = 2.92E-16), and dendritic cells (R = 0.488, *p* = 1.54E-60), whereas it was negatively correlated with the infiltration of CD8^+^ T cells (R = –0.028, *p* = 3.75E-01) and macrophages (R = –0.066, *p* = 3.68E-02) (Figure 5C).

The correlations between SNAI1 expression and the immune cells infiltration in breast cancer subtypes were also analyzed. The SNAI1 expression level was positively related with the infiltration of CD8^+^ T cells (R = 0.1, *p* = 1.88E-01), CD4^+^ T cells (R = 0.139, *p* = 6.78E-02), macrophages (R = 0.121, *p* = 1.11E-01), neutrophils (R = 0.199, *p* = 8.50E-03), and dendritic cells (R = 0.1, *p* = 1.91E-01) in the BRCA-basal type. In the BRCA-HER2 subtype, the SNAI1 expression level was positively related with the infiltration of CD8^+^ T cells (R = 0.173, *p* = 1.47E-01), macrophages (R = 0.332, *p* = 4.33E-03), neutrophils (R = 0.052, *p* = 6.63E-01), and dendritic cells (R = 0.247, *p* = 3.67E-02). In the BRCA-luminal subtype, the SNAI1 expression level was positively related with the infiltration of CD8^+^ T cells (R = 0.235, *p* = 6.76E-08), macrophages (R = 0.245, *p* = 1.76E-08), neutrophils (R = 0.362, *p* = 1.75E-17), and dendritic cells (R = 0.279, *p* = 1.00E-10) (Supplementary Figure S7).

### Relationships Between *SNAI* Gene Copy Numbers and Immune Cell Infiltration in Breast Cancer

We conducted TISIDB analysis to unravel the relationships between *SNAI* gene copy numbers and immune cell infiltration. Correlations between *SNAI1* copy numbers and different immune cells and tumor types are visualized in heatmaps in Supplementary Figure S8A. A high *SNAI1* copy number was positively correlated with the infiltration of CD8^+^ T cells, CD4^+^ T cells, and dendritic cells, whereas it was negatively correlated with the infiltration of B cells, neutrophils and macrophage (Supplementary Figure S8B). *SNAI2* and *SNAI3* copy numbers among different immune cell types and tumor types are shown in Supplementary Figures S8C, E. The *SNAI2* copy number was positively correlated with the infiltration of CD8^+^ T cells, CD4^+^ T cells, and dendritic cells, as also found for *SNAI1* (Supplementary Figure S8D). The *SNAI3* copy number was significantly correlated with the infiltration of B cells, CD8^+^ T cells, CD4^+^ T cells, macrophages, neutrophils, and dendritic cells (Supplementary Figure S8F). These results suggested that changes in the copy numbers of *SNAI* genes reflect the infiltration of various types of immune cells in breast cancer.

Next, we analyzed SNAI alterations in multiple immune cell types. For SNAI1, arm-level deletion ranked first in B cells, CD8^+^ T cells, and neutrophils. The most frequent SNAI1 alterations in immune cells were arm-level deletion and arm-level gain (Supplementary Figure S9A). For SNAI2, arm-level gain ranked first among all six immune cells analyzed (Supplementary Figure S9B). For SNAI3, arm-level deletion ranked first in B cells, CD8^+^ T cells, macrophages, neutrophils, and dendritic cells. The most frequent SNAI2 and SNAI3 alterations in different types of immune cells were arm-level deletion and arm-level gain (Supplementary Figure S9C). These results suggested that the main alteration types in SNAI genes in various immune cell types in breast cancer are arm-level deletion and arm-level gain.

### Relationships Between *SNAI* Gene Methylation and Immune Cell Infiltration

**FIGURE 6:**
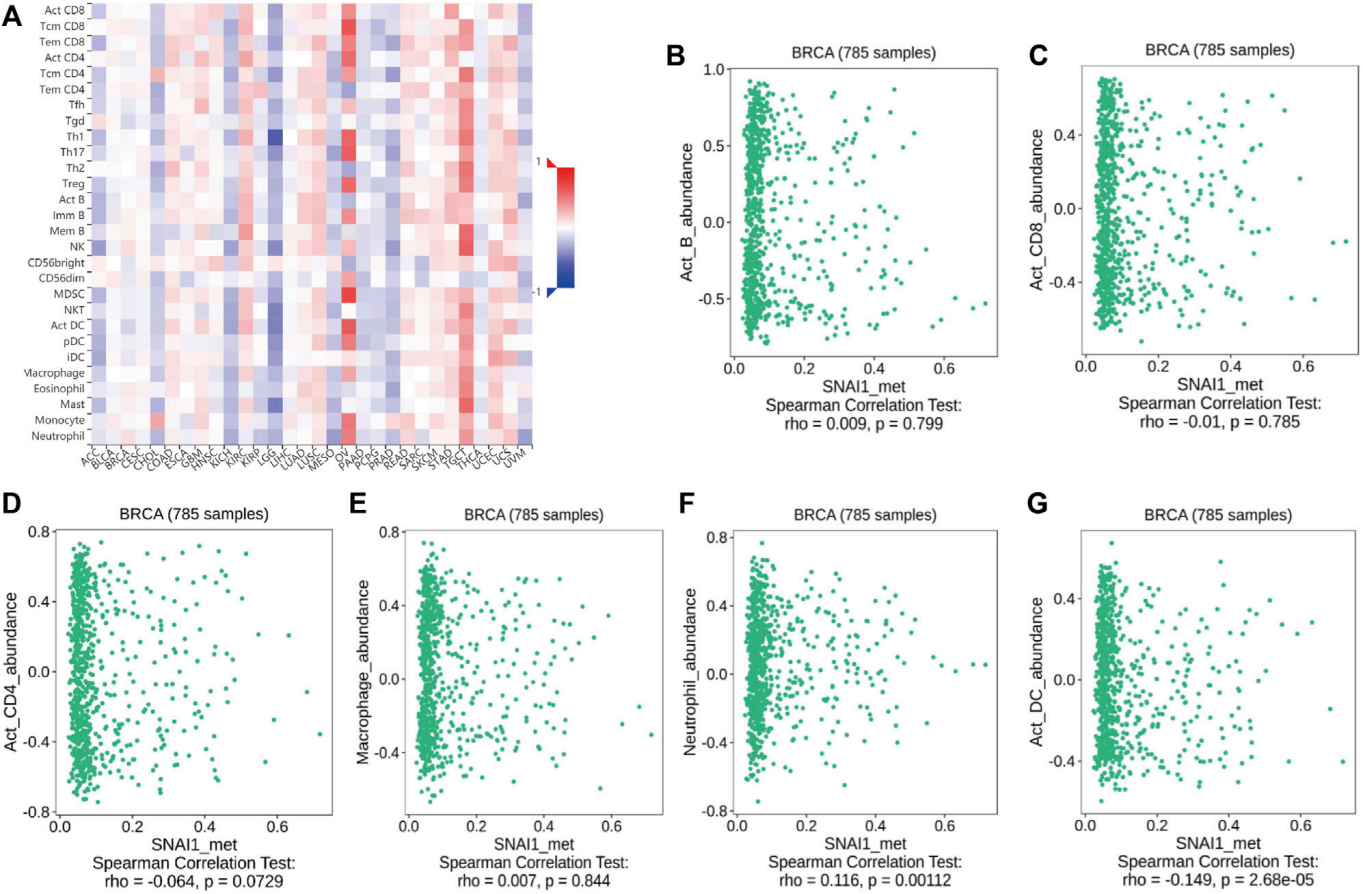
Correlations between SNAI1 methylation levels and immune cell infiltration in breast cancer **(A)** *SNAI1* methylation levels in various cancer types and immune cell types are shown in heatmaps. Correlations between changes in the *SNAI1* methylation level and the infiltration of **(B)** B cells **(C)** CD8^+^ T cells **(D)** CD4^+^ T cells **(E)** macrophages **(F)** neutrophils, and **(G)** dendritic cells in breast cancer are shown.

The above analyses revealed changes in the methylation levels of SNAI genes in breast cancer as well as the relationships between SNAI expression levels and immune cell infiltration in breast cancer. However, it remained unclear whether changes in SNAI gene methylation and immune cell infiltration are related. Therefore, we analyzed the relationships between SNAI methylation levels and the infiltration of six types of immune cells in breast cancer using TISIDB data. SNAI1 methylation levels among different immune cell types and tumor types are visualized in heatmaps (Figure 6A). SNAI1 methylation was weak positively correlated with the infiltration levels of B cells, macrophages, and neutrophils, and weak negatively correlated with the infiltration levels of CD8^+^ T cells, CD4^+^ T cells, and dendritic cells (Figures 6B–G). SNAI2 and SNAI3 methylation levels among different immune cell types and tumor types are shown in Supplementary Figures S10A, C. The SNAI2 methylation level was weak positively correlated with the infiltration levels of CD8^+^ T cells, CD4^+^ T cells, macrophages, and neutrophils, whereas it was weak negatively correlated with the infiltration levels of B cells and dendritic cells (Supplementary Figure S10B). The methylation level of SNAI3 was weak positively correlated with the infiltration levels of B cells, macrophages, and neutrophils, whereas it was weak negatively correlated with the infiltration levels of CD8^+^ T cells, CD4^+^ T cells, and dendritic cells (Supplementary Figure S10D). These results suggested that SNAI methylation levels are related to immune cell infiltration and may have opposite functions in different immune cell types. But SNAI methylation may be not a major factor in immune cell infiltration.

### Relationships Between SNAI Protein Mutations and Immune Cell Infiltration

Next, we analyzed the relationships between SNAI protein mutations and immune cell infiltration using TIMER data. The results showed SNAI1 mutation was positively correlated only with the infiltration level of neutrophils, whereas it was negatively correlated with the infiltration levels of B cells, CD8^+^ T cells, macrophages, and dendritic cells (Supplementary Figure S11A). SNAI2 mutation was positively correlated with the infiltration of macrophages, whereas it was negatively correlated with the infiltration of B cells, CD4^+^ T cells, macrophages, neutrophils, and dendritic cells (Supplementary Figure S11B). In contrast, SNAI3 mutation was positively correlated with the infiltration of CD8^+^ T cells, macrophages, neutrophils, and dendritic cells, whereas it was negatively correlated with the infiltration of B cells and CD4^+^ T cells (Supplementary Figure S11C). These results suggested that SNAI protein mutation affects immune cell infiltration and that the three SNAI proteins play different roles in immune cell infiltration.

### Immune Cell-related Survival Analysis

We used TIMER to analyze the correlations between SNAI expression levels, immune cell infiltration, and the survival rate of breast cancer patients. The results showed that patients with high SNAI1 expression and low B cell infiltration levels had a lower life expectancy than those with high SNAI1 expression and high B cell infiltration levels. Similarly, patients with high SNAI1 expression and low neutrophil infiltration levels had a poor prognosis. However, patients with high SNAI1 expression and low CD8^+^ T cell, CD4^+^ T cell, macrophage, or dendritic cell infiltration levels had a longer life expectancy than those with high SNAI1 expression and high immune cell infiltration levels (Supplementary Figure S12A). Patients with high SNAI2 expression and high CD8^+^ T cell, macrophage, neutrophil, or dendritic cell infiltration levels had a lower life expectancy than those with high SNAI2 expression and high immune infiltration levels, whereas patients with high SNAI2 expression and high B cell or CD4^+^ T cell infiltration levels had a longer life expectancy than those with high SNAI2 expression and high immune cell infiltration levels (Supplementary Figure S12B). Patients with low SNAI3 expression and high B cell, CD8^+^ T cell, macrophage, neutrophil, or dendritic cell infiltration levels had a lower life expectancy than those with low SNAI3 expression and low immune infiltration levels (Supplementary Figure S12C). These results suggested that high immune cell infiltration levels in patients with high SNAI1 and SNAI2 expression are associated with a bad prognosis, whereas SNAI3 in relation with high immune cell infiltration has the opposite effect and is related with a good prognosis.

### Correlations Between SNAI Expression Level and Immunomodulator Expression in Breast Cancer

**FIGURE 7:**
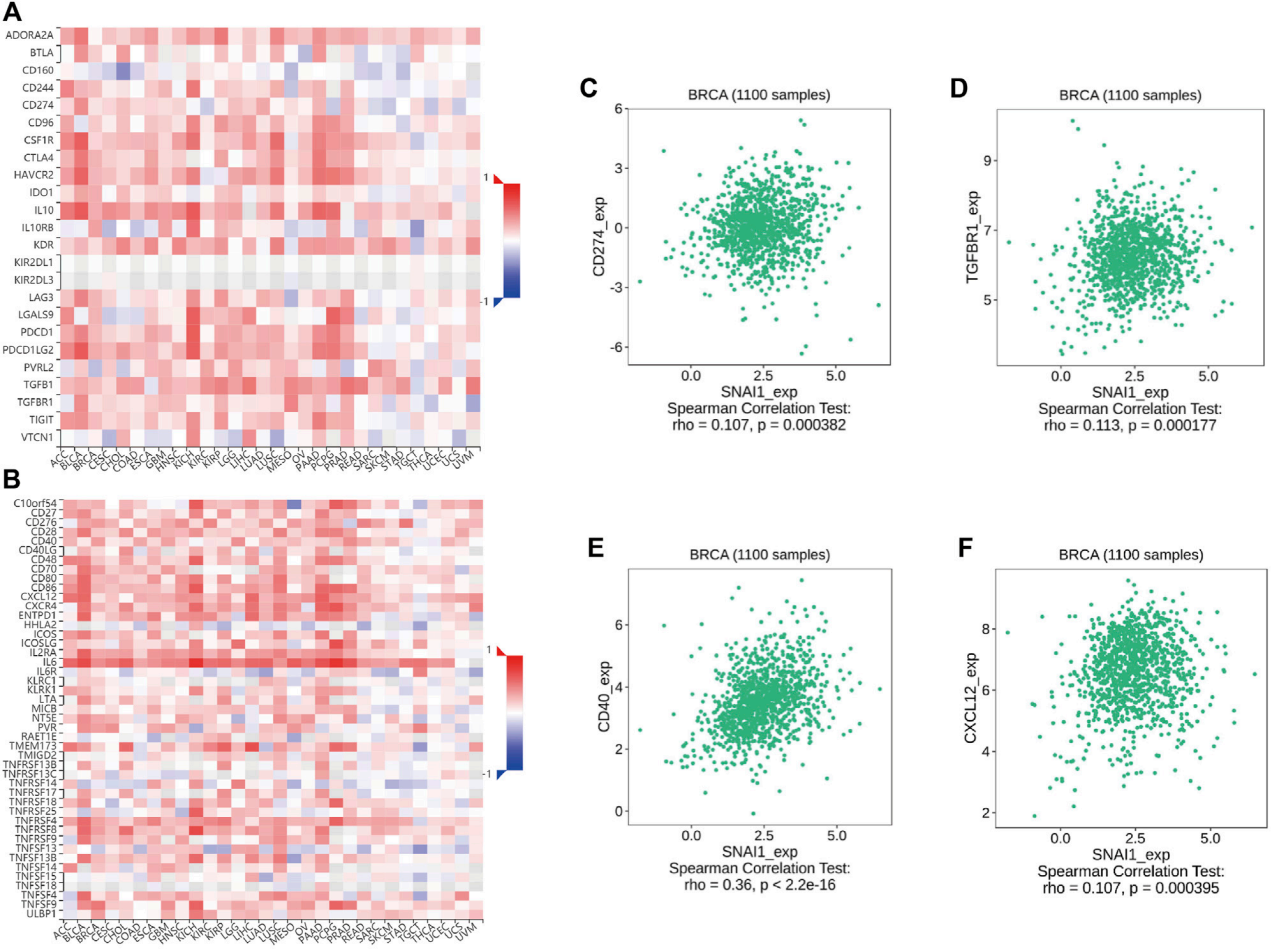
Correlations between differentially expressed SNAI1 and the expression levels of immuno-inhibitors and immunostimulators in breast cancer **(A)** Correlations between SNAI1 expression levels in various cancer types and various immuno-inhibitors are shown in heatmaps. Correlations between SNAI1 expression levels and the expression levels of **(B)** CD274 and **(C)** TGFBR1 in breast cancer are shown **(D)** Correlations between SNAI1 expression levels in various cancer types and immunostimulators are shown in heatmaps. Correlations between SNAI1 expression levels and the expression levels of **(E)** CD40 and **(F)** CXCL12 in breast cancer are shown.

Immunomodulators are divided into immuno-inhibitors and immunostimulators. It is currently unclear whether immunomodulators are related to SNAI expression. Using TISIDB data, we found the correlations between SNAI expression levels and immunomodulator expression in breast cancer. The correlations between SNAI1 expression levels and immuno-inhibitors and immunostimulators are visualized in heatmaps (Figures 7A,B). The results showed that the expression of CD274 and TGFBR1, which are immuno-inhibitors, was correlated with the SNAI1 expression level (Figures 7C,D). The expression of CD40 and CXCL12, which are immunostimulators, were also correlated with the SNAI1 expression level (Figures 7E,F). The correlations between SNAI2 and SNAI3 expression levels and immuno-inhibitors and immunostimulators are shown in Supplementary Figures S13A,B, G,H, respectively. CD274, TGFBR1, CD40, and CXCL12 expression was correlated with the SNAI2 expression level (Supplementary Figures S13C–F). CD274, CD40, and CXCL12 expression was positively correlated with the SNAI3 expression level, whereas that of TGFBR1 was negatively correlated with the SNAI3 expression level (Supplementary Figures S13I–L). These results suggested that SNAI expression affects the expression of immunomodulators, which may influence immune system function in breast cancer.

### Correlations Between SNAI Expression Levels and Chemokine Expression in Breast Cancer

The chemokine family plays a vital role in controlling the migration and residence of immune cells through receptor recognition. However, it remains unclear whether chemokines and receptors are related to SNAI expression levels. Using TISIDB data, we analyzed the correlations between SNAI expression levels and chemokine expression levels. The correlations between SNAI1 expression and chemokine and receptor levels are visualized in heatmaps (Supplementary Figures S14A,B). The expression of the chemokines CCL2 and CCL5 was positively correlated with the SNAI1 expression level (Supplementary Figures S14C,D). The expression of the receptors CCR5 and CCR7 was also positively correlated with the SNAI1 expression level (Supplementary Figures S14E,F). The correlations between SNAI2 and SNAI3 expression level and chemokines and receptors are shown in Supplementary Figures S14G,H, M,N, respectively. The expression of CCL2, CCL5, CCR5, and CCR7 was positively correlated with the SNAI2 expression level (Supplementary Figures S14I–L). The expression of CCL2, CCL5, CCR5, and CCR7 was positively correlated with the SNAI3 expression level (Supplementary Figures S14O–R). These results suggested that SNAI expression affects the expression of chemokines and their receptors, which may influence immune cell activation and killing power in breast cancer.

### Correlations Between Drug Sensitivity and SNAI Expression Levels

**FIGURE 8:**
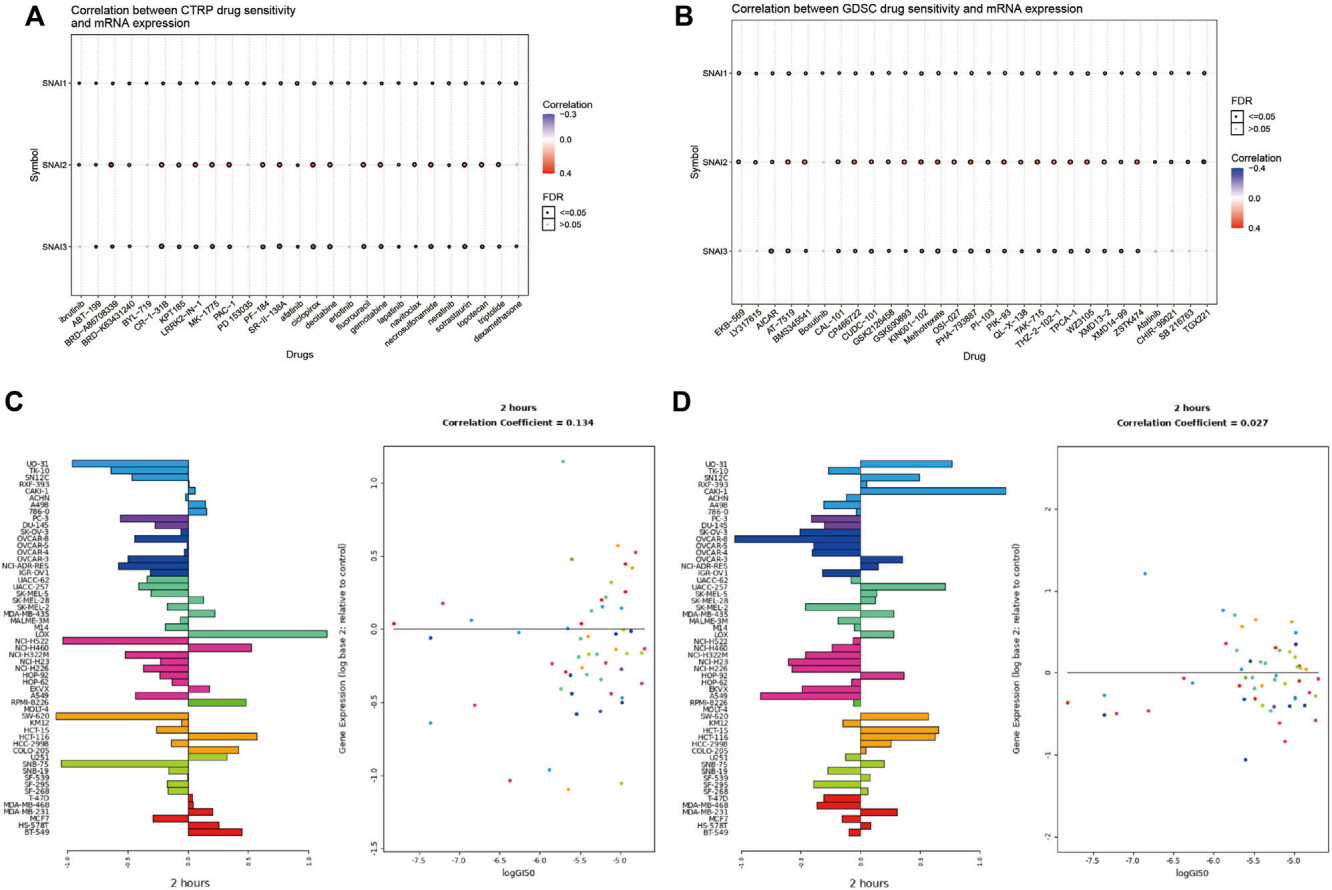
Correlations between drug sensitivity and SNAI expression levels **(A)** Correlations between drug sensitivity and *SNAI* mRNA expression based on CTRP data **(B)** Correlations between drug sensitivity and *SNAI* mRNA expression based on GDSC data **(C)** SNAI1 expression levels after lapatinib treatment for 2 h in various cancer cell lines **(D)** SNAI2 expression levels after lapatinib treatment for 2 h in various cancer cell lines.

The above analyses revealed the relationships between SNAI family members and tumors. Finally, we analyzed whether SNAI family members may serve as therapeutic targets for inhibiting tumor development. We used drug databases to analyze drugs related to *SNAI* expression levels. The 30 drugs for which we found the strongest correlations between *SNAI* gene expression and drug sensitivity in pan-cancer in the CTRP and GDSC databases are shown in bubble charts in Figures 8A,B. For most drugs, drug sensitivity increased with increasing *SNAI2* expression. Next, we analyzed the effect of lapatinib, a commonly used antitumor drug, on SNAI expression in different types of cancer cells. The results showed that after treatment with lapatinib for 2 h, the *SNAI1* and *SNAI2* expression levels in most tumor cell types were decreased, as was their survival ability (Figures 8C,D). These results suggested that SNAI family members have a strong effect on tumors and may serve as therapeutic targets for the development of new antitumor drugs.

## Discussion

The onset and development of breast cancer is complex and involves numerous genetic and environmental factors (Polyak, 2007). An increasing number of studies show immune cell infiltration varies widely and affects patient prognosis. Infiltrating immune cells affect tumor development and metastasis through different signaling pathways. Further, numerous gene alterations have been identified by comparing tumor and normal tissues, and *SNAI* family members show a high mutation frequency. In mammals, the SNAI family comprises SNAI1–3. However, the clinical relevance of immune cells and related SNAI expression in breast cancer remains unclear.

In the current study, we analyzed the effects of the SNAI family members on tumors and their relationships with survival outcome by probing various public databases. The results indicated that SNAI1 and SNAI2 are highly expressed in breast cancer and are positively correlated with poor prognosis. However, the SNAI3 expression had an opposite effect on the prognosis of breast cancer patients. These results suggest that SNAI3 has an opposite role to SNAI1 and SNAI2 in the onset and development of breast cancer. SNAI1 is one of the key proteins of EMT and is closely related to E-cadherin (Cano et al., 2000). Thus, it may serve as a prognostic factor in various tumors. Studies have demonstrated that SNAI1 is a tumor-promoting factor. Our data corroborated that SNAI may serve as a prognostic biomarker in breast cancer. SNAI3 has been less well studied, but we found that it is positively correlated with good prognosis in breast cancer. A clinical study showed that the decrease of SNAI1 expression level will lead to the increase of CDH1 expression level, thereby promoting cell migration, invasion, and poor prognosis (Chen et al., 2018). The finding is consistent with our results and corroborates that SNAI3 can inhibit tumor onset and development. Our results provide a clinical basis for follow-up research. Epigenetics plays an important role in the onset and development of breast cancer (Wu et al., 2015). It has been reported that gene promoter methylation is frequent in breast cancer. Consistent herewith, *SNAI3* promoter methylation was increased, resulting in decreased *SNAI3* expression. We comprehensively analyzed methylation changes in *SNAI* genes. *SNAI* methylation changes may serve as biomarkers in breast cancer from these findings. Loss or mutation of *TP53* is the most common genetic lesion in cancer (Petitjean et al., 2007; Hainaut and Pfeifer, 2016). In the current study, we found that patients with *TP53* mutation tended to have high SNAI1 expression (Lee et al., 2009). This result suggests that *SNAI1* and *TP53* mutation are positively correlated, which indicates that *SNAI1* is correlated with the degree of malignancy of tumors.

SNAIs have critical roles in regulating immune cell. For example, SNAIs induce regulatory T cells or tumor-associated macrophages which belong to immunosuppressive cells (Kudo-Saito et al., 2009; Hsu et al., 2014). In ovarian cancer, they induce intratumoral trafficking of myeloid-derived suppressor cells by upregulating CXCR2 ligands (Taki et al., 2018). It is important to analyze the correlations between SNAI expression levels and the infiltration of different immune cell types. TIMER data was used to analyze the correlations between SNAI expression levels and the infiltration of immune cells. We found that high SNAI1 expression was significantly associated with increased in infiltration of CD8^+^ T cells, CD4^+^ T cells, and dendritic cells. Further, we analyzed the relationships between SNAI mutations and immune cell infiltration. To explore how SNAI affects the infiltration of immune cells, we analyzed the relationships between SNAI family members and immunomodulator and chemokine expression. The chemokine CXCL5 enhances the activation of Snail causing EMT to induce invasion of colorectal cancer (Zhao et al., 2017). IL-25 is negatively correlated with patient prognosis by inducing alternative activation of macrophages promoted tumorigenesis by increasing SNAIL expression (Li et al., 2019). We found that numerous immunomodulators and chemokines are positively correlated with SNAI expression in breast cancer. This finding provides a clinical basis for further experimental verification.

Finally, we found that certain drugs affect the expression of SNAI family members and thus may affect targeted tumor therapies. In future, we can screen small-molecule compounds that affect SNAI expression to develop new drugs for tumor treatment.

In conclusion, the current study deepened our understanding of the roles of SNAI family members and their relationships with immune cells in breast cancer. This study provided a basis for elucidating the molecular mechanisms of SNAIs in the onset and development of breast cancer and provided a potential therapeutic target for treating breast cancer.

## Data Availability

The datasets presented in this study can be found in online repositories. The names of the repository/repositories and accession number(s) can be found in the article/Supplementary Material.
