# Supplementary material for: Analysis of the Effect of SNAI Family in Breast Cancer and Immune Cell
Source: Front Cell Dev Biol. 2022 Jul 8;10:906885. doi: 10.3389/fcell.2022.906885 (PMC9309217; doi:10.3389/fcell.2022.906885)
Supplement: Supplementary file 2 [file Table1.DOCX]

***Supplementary Material***

**Supplementary Figure 1. Correlations between SNAI1 expression and breast cancer patient characteristics.** SNAI1 expression levels in breast cancer patients classified by **(A)** patient ethnicity, **(B)** histologic subtype, **(C)** breast cancer subclass, and **(D)** *TP53* mutation status.

**Supplementary Figure 2. *SNAI* gene alterations in cancer. (A)** Proportions of the various *SNAI* alterations analyzed in cancer patients. **(B)** Genetic alterations in *SNAI*, *SNAI2*, and *SNAI3* in breast cancer patients.

**Supplementary Figure 3. Locations of the mutations in the *SNAI* genes in cancer. (A)** Locations of mutations along the *SNAI1*, *SNAI2*, and *SNAI3* genes in various cancers. **(B)** Positions of SNAI1, SNAI2, and SNAI3 mutations on the protein structure.

**Supplementary Figure 4. Analysis of differentially expressed genes (DEGs) in correlation with SNAI2 in breast cancer. (A)** Volcano plot showing the up- and downregulated genes correlated with SNAI2 expression (Pearson test). Significantly positively correlated **(B)** and negatively correlated **(C)** genes are shown in heatmaps. **(D)** Kyoto Encyclopedia Genes and Genomes (KEGG) pathway analysis of the significant DEGs in correlation with SNAI2. **(E)** Gene ontology (GO) analysis of the significant DEGs in correlation with SNAI2.

**Supplementary Figure 5. Analysis of DEGs in correlation with SNAI3 in breast cancer. (A)** Volcano plot showing the up- and down-regulated genes correlated with SNAI3 expression (Pearson test). Significantly positively correlated **(B)** and negatively correlated **(C)** genes are shown in heatmaps. **(D)** KEGG pathway analysis of the significant DEGs in correlation with SNAI3. **(E)** GO analysis of the significant DEGs in correlation with SNAI3.

**Supplementary Figure 6. Correlations between *SNAI1* methylation levels and breast cancer patient characteristics.** SNAI1 methylation levels in breast cancer patients classified by **(A)** cancer stage, **(B)** histological subtype, **(C)** patient ethnicity, and **(D)** breast cancer subclass are shown.

**Supplementary Figure 7. Correlations between SNAI1 expression and immune cell infiltration in various breast cancer subtypes.** Correlations between SNAI1 expression and the infiltration of B cells, CD8^+^ T cells, CD4^+^ T cells, macrophages, neutrophils, and dendritic cells in BRCA-basal, BRCA-HER2, and BRCA-LumA breast cancer subtypes.

**Supplementary Figure 8. Correlations between *SNAI* gene copy numbers and immune cell infiltration in breast cancer.** *SNAI1* (**A)**, *SNAI2* (**C)**, and *SNAI3* (**E)** gene copy numbers in various cancer types and immune cell types are shown in heatmaps. Correlations between *SNAI1* (**B)**, SNAI2 (**D)**, and *SNAI3* (**F)** gene copy number changes and infiltration levels of B cells, CD8^+^ T cells, CD4^+^ T cells, macrophages, neutrophils, and dendritic cells in breast cancer are shown.

**Supplementary Figure 9. Correlations between *SNAI* gene deletion or amplification with immune cell infiltration in breast cancer.** Correlations between deletion or amplification of *SNAI1* (**A)**, *SNAI2* (**B)**, and *SNAI3* (**C)** and infiltration levels of B cells, CD8^+^ T cells, CD4^+^ T cells, macrophages, neutrophils, and dendritic cells in breast cancer are shown.

**Supplementary Figure 10. Correlations between *SNAI2* and *SNAI3* methylation levels and immune cell infiltration in breast cancer.** *SNAI2* (**A)** and *SNAI3* (**C)** methylation levels in various cancer types and immune cell types are shown in heatmaps. Correlations between changes in the methylation levels of *SNAI2* **(B)** and *SNAI3* **(D)** and the infiltration of B cells, CD8^+^ T cells, CD4^+^ T cells, macrophages, neutrophils, and dendritic cells in breast cancer are shown.

**Supplementary Figure 11. Correlations between *SNAI* mutations and immune cell infiltration in breast cancer.** Correlations between the mutation frequency of *SNAI1* (**A)**, *SNAI2* (**B)**, and *SNAI3* (**C)** and the infiltration levels of B cells, CD8^+^ T cells, CD4^+^ T cells, macrophages, neutrophils, and dendritic cells in breast cancer are shown.

**Supplementary Figure 12. Prognostic value of differentially expressed SNAIs and immune cell infiltration in breast cancer.** Breast cancer patients were classified into four groups based on the median expression levels of *SNAI1* (**A)**, *SNAI2* (**B)** and *SNAI3* (**C)** and the infiltration levels of B cells, CD8^+^ T cells, CD4^+^ T cells, macrophages, neutrophils, and dendritic cells.

**Supplementary Figure 13. Correlations between differentially expressed SNAI2, SNAI3 and the expression levels of immuno-inhibitors and immunostimulators in breast cancer.** Correlations between SNAI2 **(A)** and SNAI3 **(G)** expression levels in various cancer types and various immuno-inhibitors are shown in heatmaps. Correlations between SNAI2 (**C-D**) and SNAI3 **(I-J)** expression levels and the expression levels of CD274 and TGFBR1 in breast cancer are shown. Correlations between SNAI2 **(B)** and SNAI3 **(H)** expression levels in various cancer types and various immunostimulators are shown in heatmaps. Correlations between SNAI2 (**E-F)** and SNAI3 (**K-L)** expression levels and the expression levels of CD40 and CXCL12 in breast cancer are shown.

**Supplementary Figure 14. Correlations between differentially expressed SNAIs and the expression levels of chemokines and receptors in breast cancer.** Correlations between SNAI1 **(A)**, SNAI2 (**G**), and SNAI3 **(M)** expression levels in various cancer types and various chemokines are shown in heatmaps. Correlations between SNAI1 **(C-D)**, SNAI2 (**I-J)**, and SNAI3 (**O-P)** expression levels and the expression levels of CCL2 and CCL5 in breast cancer are shown. Correlations between SNAI1 **(B)**, SNAI2 (**H**), and SNAI3 **(N)** expression levels in various cancer types and various receptors are shown in heatmaps. Correlations between SNAI1 **(E-F)**, SNAI2 (**K-L)**, and SNAI3 (**Q-R)** expression levels and the expression levels of CCR5 and CCR7 in breast cancer are shown.
