# Supplementary material for: Analysis of the Effect of SNAI Family in Breast Cancer and Immune Cell
Source: Front Cell Dev Biol. 2022 Jul 8;10:906885. doi: 10.3389/fcell.2022.906885 (PMC9309217; doi:10.3389/fcell.2022.906885)
Supplement: Supplementary file 13 [file Table2.DOCX]

<https://www.jianguoyun.com/c/sd/14e6c1c/43e02a885875e5b9>
